# Comparison of safety and effectiveness of micropulse transscleral cyclophotocoagulation and “slow cook” diode laser transscleral cyclophotocoagulation in patients with refractory open-angle glaucoma

**DOI:** 10.5935/0004-2749.2023-0103

**Published:** 2024-07-09

**Authors:** Heloisa Helena Abil Russ, Regina Cele Silveira Seixas, Heloísa Andrade Maestrini, Marcos Balbino, Thatlana Almeida Pereira Fernandes, Núbia Vanessa dos Anjos Lima, Nara Lídia Vieira Lopes, Taurino dos Santos Rodrigues Neto

**Affiliations:** 1 HR Oftalmologia, Curitiba, PR, Brazil; 2 HCLOE Clínica de Oftalmologia Especializada, São Paulo, SP, Brazil; 3 Oculare Hospital de Oftalmologia, Belo Horizonte, MG, Brazil; 4 Centro Universitário São Camilo, São Paulo, SP, Brazil; 5 VisionOne- Hospital de Olhos CBV, Brasilia, DF, Brazil; 6 Department of Ophthalmology, Universidade de São Paulo, São Paulo, SP, Brazil

**Keywords:** Sclera/surgery, Glaucoma, open-angle/surgery, Ciliary body/surgery, Intraocular pressure, Laser coagulation/methods, Lasers, semiconductor, Comparative study, Effectiveness

## Abstract

**Purpose:**

This study aimed to compare the safety and effectiveness of intraocular pressure reduction between micropulse transscleral cyclophotocoagulation and “slow cook” transscleral cyclophotocoagulation in patients with refractory primary open-angle glaucoma.

**Methods:**

We included patients with primary open angle glaucoma with at least 12 months of follow-up. We collected and analyzed data on the preoperative characteristics and postoperative outcomes. The primary outcomes were a reduction of ≥20% of the baseline value (criterion A) and/or intraocular pressure between 6 and 21 mmHg (criterion B).

**Results:**

We included 128 eyes with primary open-angle glaucoma. The preoperative mean intraocular pressure was 25.53 ± 6.40 and 35.02 ± 12.57 mmHg in the micropulse- and “slow cook” transscleral cyclophotocoagulation groups, respectively (p<0.001). The mean intraocular pressure was reduced significantly to 14.33 ± 3.40 and 15.37 ± 5.85 mmHg in the micropulse- and “slow cook” transscleral cyclophotocoagulation groups at the last follow-up, respectively (p=0.110). The mean intraocular pressure reduction at 12 months was 11.20 ± 11.46 and 19.65 ± 13.22 mmHg in the micropulse- and “slow cook” transscleral cyclophotocoagulation groups, respectively (p<0.001). The median preoperative logMAR visual acuity was 0.52 ± 0.69 and 1.75 ± 1.04 in the micropulse- and “slow cook” transscleral cyclophotocoagulation groups, respectively (p<0.001). The mean visual acuity variation was −0.10 ± 0.35 and −0.074 ± 0.16 in the micropulse- and “slow cook” transscleral cyclophotocoagulation, respectively (p=0.510). Preoperatively, the mean eye drops were 3.44 ± 1.38 and 2.89 ± 0.68 drugs in the micropulse- and “slow cook” transscleral cyclophotocoagulation groups, respectively (p=0.017), but those were 2.06 ± 1.42 and 1.02 ± 1.46 at the end of the study in the “slow cook” and micropulse transscleral cyclophotocoagulation groups, respectively (p<0.001). The success of criterion A was not significant between both groups. Compared with 11 eyes (17.74%) in the “slow cook” transscleral cyclophotocoagulation group, 19 eyes (28.78%) in the micropulse transscleral cyclophotocoagulation group showed complete success (p=0.171). For criterion B, 28 (42.42%) and 2 eyes (3.22%) showed complete success after micropulse- and “slow cook” transscleral cyclophotocoagulation, respectively (p<0.001).

**Conclusion:**

Both techniques reduced intraocular pressure effectively.

## INTRODUCTION

Glaucoma, a progressive optic neuropathy, is one of the main causes of irreversible blindness worldwide^(1)^. The treatment of refractory glaucoma includes various cyclodestructive procedures^(2)^, which reduce intraocular pressure (IOP) by damaging the secretory epithelium present in the ciliary body processes, resulting in reduced aqueous production^(3)^. Cyclophotocoagulation is one of the cyclodestructive procedures that uses a diode laser wave to induce coagulation and necrosis of the ciliary epithelium. Despite being recommended as a treatment for any type of glaucoma, cyclophotocoagulation is usually performed in more severe cases and more rarely in cases of glaucoma with good visual acuity (VA) due to the risk of complications, such as VA deterioration, hypotonia, and phthisis bulbi^(4,5,6)^.

Cyclophotocoagulation with continuous diode laser emits light from the 810 nm infrared spectrum, which is strongly absorbed by melanin. Transscleral laser targets the melanin present in the pigmented epithelium of the ciliary body, thus decreasing the rate of aqueous humor production. Traditionally, this technique delivers continuous laser energy.

Alternatives with less morbidity would be the micropulse laser cyclophotocoagulation (MP-TSCP), which uses 30% of laser energy, and the “slow cook” cyclophotocoagulation (SC-TSCP)^(7,8)^.

The MP-TSCP manages a series of short, repetitive pulses of laser energy (“on” period) separated by rest periods (“off” periods), and is different from the conventional one, which offers a continuous flow of high-intensity energy for the ciliary body. Generally, MP-TSCP uses a duty cycle of 31.3% of a 2000-mW total energy. Periods without laser activity allow thermal dissipation and cool down temperature, which could potentially reduce side effects. It targets the pars plana instead of the pars plicata, indicating that flow through the trabecular pathway possibly increases by a mechanism similar to that of pilocarpine. Because the laser does not destroy the ciliary body, the inflammatory process in the ciliary body would reduce the aqueous formation. However, its exact mechanism of action is still unknown. MP-TSCP is applied using a personalized probe that manages the laser as continuous sliding movements with energy aimed at the pars plana^(9,10,11)^. Conversely, SC-TSCP delivers a continuous pulse of thermal energy, but with low energy and a prolonged time, which avoids the sound of ciliary body explosion, thus reducing the destructive power and inflammatory response^(9,10)^.

Most studies indicate that the efficacy of MP- and SC-TSCP would be similar to conventional transscleral photocoagulation in the treatment of refractory glaucoma, although with more consistent and predictable results. However, only a few studies compared these alternatives with lower morbidity. Therefore, this study aimed to compare the safety and effectiveness of MP- and SC-TSCP in patients with open-angle glaucoma.

## METHODS

The Institutional Review Board of HCLOE *Clinica de Oftalmologia Especializada* endorsed the study, and all data entered complied with relevant data protection and privacy guidance. The patient’s identification remained anonymous throughout the study. The research methods satisfied the Helsinki Declaration. The study did not require informed consent due to its retrospective design and noninterventional review of medical records.

This is a longitudinal retrospective cohort, multi-center study. By reviewing the charts, we identified patients with moderate to advanced primary open-angle glaucoma (POAG) who were treated with transscleral cyclophotocoagulation using the MP- or SC-TSCP technique and who had at least 12 months of follow-up. All surgeons performed the techniques, and patients who met the criteria for inclusion were considered from 2017 to 2021. All patients had uncontrolled IOP despite maximally tolerable antiglaucoma medications. We included one eye from each patient diagnosed with POAG and treated with MP- or SC-TSCP. Moreover, we excluded those who underwent any other ocular surgery, including outpatient laser treatment, such as trabeculoplasty, in the 3 months before being considered for this study.

Different glaucoma specialists treated patients from four different sites (HCLOE Opty Group Brazil-São Paulo, SP; Oculare Hospital de Oftalmologia-Belo Horizonte, MG; HR Oftalmologia-Curitiba, PR; and CBV Hospital de Olhos-VisionOne).

Data on preoperative characteristics, surgical procedure(s), and postoperative outcomes were collected and analyzed.

SC-TSCP used a diode laser (810 nm) (Model Oculight SLx, Iris Medical Instruments Inc., Mountain View, California, USA) after peribulbar anesthesia with 5-7 ml of 0.5% bupivacaine and was performed by experienced specialists in glaucoma. The G probe was positioned 1.5 mm from the limbus, and the initial power was 1500 mW for 4 s at >270° while sparing the 3’ and 9 o’clock positions. Power was titrated down or up, but never exceeding 1800 mW, to avoid any sound of ciliary body explosion. MP-TSCP was performed using 1R1DEX Cyclo G6 (Glaucoma Laser System, Mountain View, CA, USA), and the first-generation probe was used.

The treatment settings were 2000 mW at 31.3% duty power applied to the superior and inferior hemispheres for 240-360 s each. The MP3 probe was applied perpendicularly 2 mm from the limbus on the adjacent sclera over a viscoelastic layer or anesthetic gel. The sweeping technique consisted of a slow, continuous sliding motion in two arcs of 180° superior and inferior, avoiding the 3 and 9 o’clock positions.

After the procedure, patients received a fixed combination of corticosteroids (prednisolone) and antibiotics (gatifloxacin) every 2-3 h in the first week, which was gradually reduced for the next 30 days, and atropine 1% every 12 h for 2 weeks.

Follow-up consisted of IOP analysis, number of topical medications, intra- and postoperative complications, VA, need for additional glaucoma surgery, and need for repeat MP- or SC-TSCP.

Success was defined as a decrease of >20% of preoperative IOP (criterion A) and/or IOP measuring between 6 and 21 mmHg (criterion B), without medication (complete success) and with medication (qualified success). Failure was defined as when the criteria were not met in any visit. Moreover, failure was also considered for the need for any other antiglaucoma surgery to reduce IOP, such as fistulizing surgeries, drainage implants, or traditional cyclodestructive procedures, after MP- or SC-TSCP. However, we did not consider multiple MP- or SC-TSCP procedures. All repeated MP- and SC-TSCP procedures followed the same parameters as the initial procedure.

STATA Statistical Package version 13 for Windows (Stata Corp, College Station, USA) was used for data analysis and statistics. Categorical variables were presented as numbers and percentages and were analyzed using the x^2^ or Fisher’s exact test. Continuous variables were expressed as mean and standard deviation or median and interquartile range (IQR). Normality was analyzed using the skewness and Kurtosis tests. Student’s T and Wilcoxon rank-sum tests were used for normally distributed and asymmetrical continuous variables, respectively. To compare survival curves between groups (Kaplan-Meier), log-rank and Cox regression tests are used if any covariable is included in the comparisons. A p<0.05 was considered statistically significant.

## RESULTS

A total of 128 eyes underwent the procedures, with 66 and 62 eyes receiving MP- and SC-TSCP, respectively. The demographics of the two groups did not differ significantly: mean age of 66.1 ± 12.78 and 66.2 ± 16.03 years in the MP- and SC-TSCP groups, respectively (p = 0.490). The MP- and SC-TSCP groups were composed of 26 and 33 female patients, as well as 40 and 29 male patients, respectively (p=0.120). However, VA was different between the two groups, with mean preoperative VA (logMAR) of 0.52 ± 0.69 and 1.75 ± 1.04 in the MP- and SC-TSCP groups, respectively (p<0.001).

Both groups underwent several previous surgical procedures. The SC-TSCP group had a greater number of patients undergoing previous trabeculectomy and needling. However, other procedures showed no statistical difference between the two groups (Table 1). The total amount of energy was 213.22 ± 45.01 and 143.94±30.22 J in the MP- and SC-TSCP groups, respectively (p=0.001).

**Table 1:** Number of previous surgical procedures

Procedures n (%)	Slow cook	Micropulse	p-value
**SLT or ALT**			
1	3	2	0.504
*2*	1	0	
Total	4	2	
**Vitreous injection**			
1	2	0	0.103
2	2	0	
3	0	2	
Total	4	2	
**Cataract (phaco or ECCE)**			
1	41	47	0.535
Total	41	47	
**Refractive surgery**			
1	1	1	0.96
Total	1	1	
**Corneal transplant**			
1	3	4	0.13
3	1	1	
Total	4	5	
**Retinal surgery**			
1	4	5	0.13
2	4	0	
3	1	0	
Total	9	5	
**Trabeculectomy or sclerotomy**			
1	26	16	0.001
2	10	2	
3	1	0	
Total	37	18	
**Needling**			
1	5	10	0.003
2	11	2	
3	5	1	
4	4	0	
5	0	1	
Total	25	14	
**Tube implant**			
1	9	5	0.209
Total	9	5	
**MIGS**			
2	1	1	0.96
Total	1	1	
**Previous cyclophotocoagulation**			
1	1	0	0.30
Total	1	0	
**Revision trabeculectomy**			
1	8	3	0.092
Total	8	3	

### Effect of each technique on IOP

**Figure 1:**
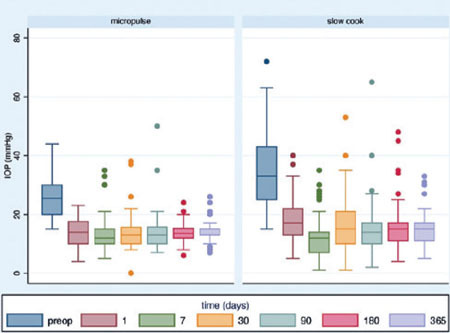
Intraocular pressure variation across time in the micropulse and slow cook laser groups.

The mean IOP at presentation was significantly greater in the SC-TSCP group. The initial IOP was 25.53 ± 6.40 and 35.02 ± 12.57 mmHg in the MP- and SC-TSCP groups, respectively (p<0.001). The mean IOP was reduced significantly to 14.33 ± 3.40 and 15.37 ± 5.85 mmHg in the MP- and SC-TSCP at 12 months, respectively (Figure 1) (p=0.110), accounting for 43.87% and 56.11% mean IOP reduction at 12 months, respectively (p=0.002).

### Effect of each technique on antiglaucoma drugs

**Figure 2:**
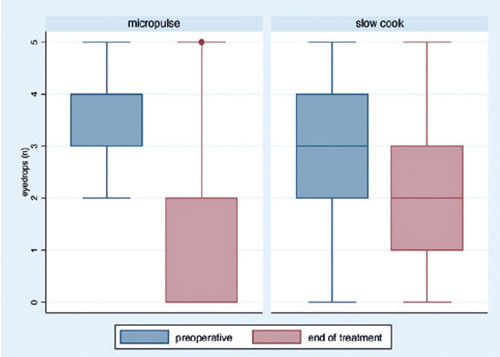
Eyedrops variation across time in the slow cook and micropulse laser groups.

The mean preoperative eye drops used were 3.44 ± 1.38 and 2.89 ± 0.68 drugs in the MP- and SC-TSCP groups, respectively (p=0.017). The mean eye drops used at the end of the study were 2.06 ± 1.42 and 1.02 ± 1.46 in the SC- and MP-TSCP groups, respectively (p<0.001). The eyedrops reduction variation was 2.38 ± 1.55 and 0.82 ± 1.686 in the MP- and SC-TSCP groups, respectively (p<0.001; Figure 2).

### Success rate, retreatment, and complications

**Figure 3:**
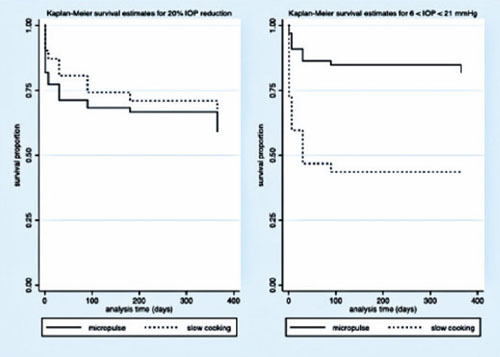
Kaplan-Meier curve representing the probability of treatment success over time. Criterion A: 20% IOP reduction; Criterion B: 6 < IOP < 21 mmHg.

**Figure 4:**
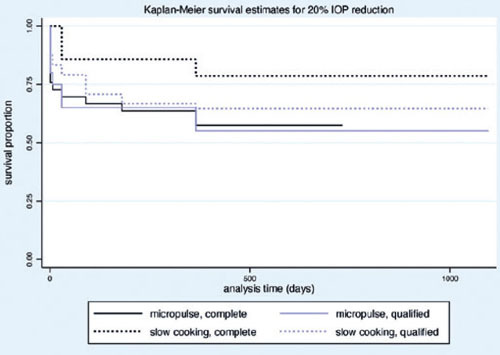
Kaplan-Meier curve representing the probability of complete and qualified success of 20% IOP reduction (criterion A) over time.

The mean VA variation (logMAR) was -0.10 ± 0.35 and -0.074 ± 0.16 in the MP- and SC-TSCP groups, respectively (p = 0.510). At the end of the study, the mean VA (logMAR) was 0.56 ± 0.70 and 1.85 ± 1.07 in the MP- and SC-TSCP groups, respectively (p<0.001). The success of criterion A at 12 months was not significant between both groups. Complete success was achieved in 19 eyes (28.78%) and 11 eyes (17.74%) following MP- and SC-TSCP, respectively (p=0.171). Qualified success was achieved in 11 (16.66%) and 31 (50%) eyes following MP- and SC-TSCP, respectively (p = 0.460). The number of patients achieving Criterion B (IOP between 6 and 21 mmHg) was significantly different. Complete success was achieved in 28 (42.42%) and 2 eyes (3.22%) following MP- and in SC-TSCP, respectively (p<0.001), whereas qualified success was achieved in 16 (24.24%) and 23 eyes (37.09%) following MP- and SC-TSCP, respectively (p = 0.015). Figure 3 shows the Kaplan-Meier (KM) curves of criteria A and B. Figures 4 5 show the Kaplan-Meier plot for the probability of complete and qualified success of criteria A and B, respectively. A retreatment session was necessary for 8 (14.03%) and 15 eyes (24.19%) in the MP- and SC-TSCP groups, respectively. Only one patient in each group needed a third application. Table 2 shows the postoperative complications. The most frequent complication was corneal decompensation, with 2 and 3 cases in the MP- and SC-TSCP groups, respectively. The incidence of phthisis was 1.61% only in the SC-TSCP group.

**Figure 5:**
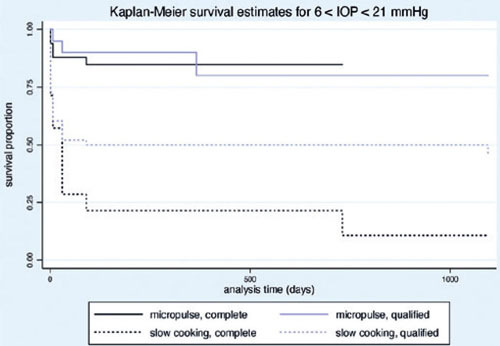
Kaplan-Meier curve representing the probability of complete and qualified success of IOP between 6 and 21 mmHg (criterion B) over time.

**Table 2:** Complications reported in micropulse and slow cook laser groups at 12 months of follow-up

Variable	Micropulse	Slow cook
Corneal de-epithelialization	1	0
Conjunctivitis	1	1
Corneal decompensation	2	3
Visual acuity worsening - 2 lines	0	2
Loss of luminous perception	0	3
Phthisis	0	1
Macular edema	0	5
Others	5	0
Total	9	15

p=0.006.

## DISCUSSION

Traditionally, cyclodestructive procedures have been considered as treatment for refractory glaucoma in eyes with poor VA potential, as well as blind and painful eyes associated with high IOP^(11)^. These procedures were reserved for these specific cases because of possible significant complications, such as pain, prolonged inflammation, and phthisis^(12)^. Recent literature reviews indicate that diode lasers with a minimally invasive energy intervention can potentially significantly reduce IOP and offer a favorable complication profile in managing refractory glaucoma cases^(13,14)^.

Our study showed that both procedures markedly decreased IOP in patients with refractory glaucoma with uncontrolled IOP despite maximal tolerated medical treatment. The mean IOP decreased by 43.86% and 56.11 % in the MP- and SC-TSCP group in 1 year, respectively. Moreover, >50% of both groups achieved at least a 20% IOP reduction after 1 year. Patients who had IOP control without eye drops at the end of treatment were higher in the MP-TSCP group (70.21% vs 29.79% in the MP- and SC-TSCP groups, respectively). The number of glaucoma medications was significantly reduced in both groups.

When 20% IOP reduction from baseline was used as a success criterion, both groups maintained the reduction throughout the study period, with no difference between groups. Notably, the SC-TSCP group had a significantly higher initial IOP (35.02 mmHg), which justifies the significant difference in the numbers of patients achieving IOP between 6 and 21 mmHg at 12 months (81.81% vs 40.32% in the MP-and SC-TSCP groups, respectively). The higher mean initial IOP in the SC-TSCP group may also reflect greater disease severity. Based on their MD and VA, future studies including POAG patients with less severe disease should be conducted to better compare SC-TSCP with MP-TSCP results.

Regarding the efficacy of MP-TSCP on IOP lowering, our success rates are lower than previously published MP-TSCP studies, which reported mean IOP reductions of 63% with a mean follow-up of 6-18 months^(15,16,17,18,19,20,21)^. Previous SC-TSCP studies reported a mean IOP reduction of 41.45%-54.8%^(7,22,23,24)^. This variability might be explained by differences in the glaucoma types and success criteria in each study, as well as the laser settings, of which there is no established consensus. Better outcomes were reported in studies using higher energy levels, but the incidence of complications can be also higher^(19,25)^. Additionally, the inclusion of patients with neovascular glaucoma in other studies increases results variability because they provide various and unpredictable results.

In POAG cases, MP-TSCP provides a very satisfactory safety profile, with low morbidity, and IOP stability over 12 months, as well as the lower need for additional medication. Similar to other studies, VA did not worsen in patients who underwent the procedures, which expands the range of indication opportunities, not only in cases of refractory glaucoma. These success rates were consistent with prior studies with lower rates of postoperative complications^(15,26)^. Previous studies reported that using energy between 112 and 150 J obtained a moderate IOP decrease of approximately 35% for up to 15 months with few complications. Energy levels <100 J caused no side effects but yielded lower IOP reductions and shorter survival of effect. 1n contrast, IOP was greatly reduced with energy levels >200 J (320 s × 2000 mW × 31.3% duty cycle), but severe complications were more prevalent^(10)^. Fluence, which includes sweep velocity, may have shown a closer relationship with the IOP-lowering effect than total energy. A relationship between fluence and the number of sweeps (which generates total treatment time) may be the optimal combination of variables to measure overall energy delivery to the eye. Accurately measuring overall energy delivery to the eye may allow for proper dosimetry to achieve a sustainable IOP-lowering effect while maximizing the safety profile^(27)^.

SC-TSCP is one of the emerging relatively noninvasive interventions that can be used in different patient populations. A recent research evaluating the outcomes of SC-TSCP as an initial surgical treatment showed that IOP decreased from 27.5 ± 9.8 preoperatively to 16.1 ± 6.3 mm Hg postoperatively, with a mean percentage IOP reduction of 42.1% and 75.7% of eyes with ≥20% decrease in their baseline IOP^(7)^. This was comparable to our findings wherein the mean percentage IOP reduction was 56.11% and 67.74% of the patients with ≥20% reduction from pretreatment IOP.

The SC-TSCP group presented more patients who underwent previous glaucoma surgeries, such as trabeculectomy and needling, as well as worse VA and higher IOP at the beginning of the study. This indicates that although both groups were composed of patients with POAG of similar age, the SC-TSCP group probably had more advanced disease, making direct comparisons between groups more difficult.

Our study showed a small percentage of serious complications, such as loss of luminous perception in 4.83% and phthisis in 1.61% only in the SC-TSCP. However, it is difficult to conclude whether they are due to only the procedure or extreme severity of the cases and the sum of all previous procedures, which certainly contributes to unfavorable developments in this group. Another complication was macular edema, which was found in 8.06% of the SC-TSCP group only, although all cases had good responses with one drop, four times daily for a month of non-steroidal anti-inflammatory eye drops.

The most common complication was corneal decompensation (4.83% and 3.03% in the SC- and MP-TSCP groups, respectively). This complication may also be due to the cumulative effect of previous treatments and the persistently very high IOP before cyclophotocoagulation, which represents significant stress on the corneal endothelium.

We did not encounter other serious complications described in the literature, such as sympathetic ophthalmia, malignant glaucoma, scleritis, and ocular perforation.

Factors possibly associated with corneal decompensation include episodes of glaucoma with high pressure, inflammation, diabetes, cornea guttata, use of high-laser energy, history of several previous surgeries, and performing the procedure very close to the limbus^(28)^. To document corneal endothelium health before the procedure, specular microscopy could help predict and thereby forewarn the patient of possible corneal complications.

Our study was limited by the relatively small number of subjects and the severity of the SC-TSCP cases. Because of its retrospective nature, patients were not randomized to MP- and SC-TSCP, which may have led to selection bias. The technique was chosen based on the laser availability in each center; thus, no specific identifiable variable predisposed a surgeon to choose one treatment over another. A future study including patients with POAG with less severe disease based on their MD and VA should be conducted to better compare SC-TSCP with MP-TSCP results. We also suggest monitoring the patients for longer a period because 1-year follow-up is a relatively short period to evaluate complications of procedures involving cyclodestruction.

In conclusion, MP- and SC-TSCP achieved a relatively good efficacy/safety compromise for refractory glaucoma in the medium term, including patients with high IOP. To date, determining which patients preoperatively could have a better response to treatment (predictive factors) is not yet possible. The SC-TSCP provided the highest mean IOP reduction, and the MP-TSCP provided minimal ocular complications. This safety profile may make MP-TSCP be used earlier in the treatment of glaucoma. The two techniques of diode laser delivery demonstrated efficient IOP reduction from baseline with lower complication rates, particularly MP-TSCP. To prevent the risk of late hypotony and phthisis, starting with mild treatment parameters and taking care of poor endothelium health, gross polymorphism, and pleomorphism with a low endothelial cell count in the specular microscopy would be reasonable options.
